# Complications and radiographic changes after implantation of interspinous process devices: average eight-year follow-up

**DOI:** 10.1186/s12891-023-06798-9

**Published:** 2023-08-23

**Authors:** Kai-Yu Li, Hua-Lin Li, Lin-Jie Chen, Jian-Wei Xiang, Chen-Chao Li, Jun-Jie Weng, Nai-Feng Tian

**Affiliations:** Zhejiang Spine Research Center, Department of Spine Surgery, The Second Affiliated Hospital and Yuying, Children’s Hospital of Wenzhou Medical University, 109 Xueyuanxi Road, Wenzhou, 325000 Zhejiang China

**Keywords:** Coflex, Heterotopic ossification, Loosening, Osteolysis, Spinous process anastomosis

## Abstract

**Purpose:**

This study aims to evaluate complications, clinical outcomes, and radiographic results following Coflex implantation.

**Methods:**

We retrospectively studied 66 patients who had decompressive surgery combined with Coflex implantation to treat lumbar spinal stenosis. All imaging data were collected and examined for imaging changes. Clinical outcomes, included Oswestry Disability Index (ODI), back and leg visual analog scale (VAS) scores, were evaluated before surgery, six months after surgery and at the last follow-up. The number of complications occurring after five years of follow-up was counted. All reoperation cases were meticulously recorded.

**Results:**

66 patients were followed up for 5–14 years. The VAS and ODI scores were significantly improved compared with baseline. Heterotopic Ossification (HO) was detectable in 59 (89.4%). 26 (39.4%) patients had osteolysis at the contact site of Coflex with the spinous process. Coflex loosening was detected in 39 (60%) patients. Spinous process anastomosis was found in 34 (51.5%) patients. There was a statistically significant difference in the VAS score of back pain between patients with and without spinous process anastomosis. Nine cases of lumbar spinal restenosis were observed, and prosthesis fracture was observed in one case.

**Conclusion:**

Our study identified various imaging changes after Coflex implantation, and majority of them did not affect clinical outcomes. The majority of patients had HO, but osteolysis and Coflex loosening were relatively rare. The VAS score for back pain of these patients was higher if they have spinous process anastomosis. After five-year follow-up, we found lumbar spinal restenosis and prosthesis fracture cases.

## Introduction

Lumbar spinal stenosis (LSS) is a degenerative disease of the lumbar spine characterized by lower extremity pain or numbness, intermittent claudication, and reduced quality of life [1, 2]. If conservative treatment fails, surgical interventions such as lumbar decompression and fusion are considered the standard therapies for managing LSS [3, 4]. However, fusion surgery has been associated with various defects, including higher infection rate, bigger blood loss, longer hospital stays, and higher costs, etc. [5]. Interspinous process devices (IPDs) such as Coflex, X-Stop, and DIAM are designed and used to improve patient outcomes. IPD can keep the surgical segment dynamically stable and slows the rate of lumbar spine degeneration [6, 7].

Coflex, as an IPD, has curative effects in short- and medium-term follow-up [8]. Several studies, however, found that Coflex had a higher reoperation rate during follow-up than decompression with and without fusion [9, 10]. Furthermore, implanting Coflex in the human body may result in several unique complications such as spinous process fracture, prosthesis fracture, and device dislocation [11]. The diagnosis of these complications, and asymptomatic device failures, are often not determined solely by clinical symptoms but must be evaluated in conjunction with imaging studies. However, there are few long-term follow-up data after Coflex implantation. Currently, there is no literature describing long-term changes in Coflex, based primarily on imaging data. As a result, this study will provide detailed statistics on complications, long-term radiologic changes and clinical outcome following Coflex implantation, mainly based on imaging.

## Materials and methods

### Patient selection

Patients who underwent Coflex implantation to treat LSS at the Second Affiliated Hospital and Yuying Children’s Hospital of Wenzhou Medical University from December 2007 to December 2014 were included in the study. All these patients were followed up postoperatively for ≥ five years after the implantation. All of these patients received conservative therapy for at least three months and showed no improvement in clinical symptoms. Imaging studies such as static (anteroposterior and lateral) and dynamic (flexion and extension) radiography, CT scan, and MRI were used to confirm the diagnosis. Table 1 shows the inclusion and exclusion criteria.

**Table 1:** Summary of study inclusion and exclusion criteria

Criteria for Inclusion & Exclusion
inclusion clinical symptoms of leg or buttock pain w/ or w/o back pain CT & MRI confirmation of lumbar stenosis failed conservative treatment ≥ 3 months single level involved (L3–4, L4–5, L5–S1)
exclusion lumbar spondylolisthesis radiographic lumbar spinal instability spinal fracture, infection, deformity, tumor, or inflammatory spondylopathy concomitant serious diseases previous back surgery

### Surgical procedure

Patients were placed prone after general anesthesia was induced. Decompression and dynamic interspinous fixation were part of the procedure. A laminotomy, resection of the thickened ligamentum flavum, and an undercutting facetectomy were used in the decompressive surgery. Herniation removal was performed simultaneously in patients with disc herniation. After the interspinous ligament removal, an interspinous implant of the appropriate size was inserted into the prepared space. The clips around the spinous processes were then tightened [12]. If not contraindicated, perioperative NSAIDs were prescribed for two to four weeks. Postoperatively, patients were asked to return for a physical and imaging examination at three, six, and twelve months after surgery and every year after that.

### Radiological and clinical assessment

Radiographs were taken preoperatively and immediately postoperatively, at three months, six months, 12 months, and every year until the last follow-up. Heterotopic Ossification (HO) was classified according to current classifications in the literature as follows: Grade 0, no HO; Grade 1, with HO only in the lateral spinous process but not in the interspinous space; Grade 2, HO in the interspinous space (irrespective of the presence of Grade 1 HO), without bridging the adjacent spinous process; and Grade 3, fusion of the interspinous processes [13]. The contact site osteolysis was visible as a translucent shadow or spinous process on the plain X-ray film significantly smaller than the previous film. Coflex loosening was defined as the significant separation of Coflex device from the spinous process on extension and flexion radiographs. Spinous process anastomosis was defined as the approximation of adjacent spinous processes with or without spinous process hypertrophy.

Clinical outcomes were evaluated before surgery, six months after surgery and at the last follow-up. Primary outcome measures included Visual Analogue Scale (VAS) and Owestry Disability Index (ODI). Complications mainly included long-term complications after five years of follow-up, excluding short- and medium-term complications within five years. The diagnostic criteria for lumbar spinal restenosis were as follows: (1) presence of neurogenic intermittent claudication and pain and/or numbness in the lower extremities with or without low back pain; (2) Restenosis of the original surgical segment confirmed by MRI; and (3) a history of ineffective responses to pharmacotherapy for more than three months.

### Statistical analysis

SPSS software, version 25 (SPSS, Inc., Chicago, IL, USA), was used for statistical analysis. Dichotomous outcomes were assessed using relative risk (RR) and independent t test.

## Results

Based on inclusion and exclusion criteria, a total of 66 patients (29 males and 37 females with a mean age of 62 years) were included. The patients’ height was 165.1 ± 6.8 cm, their weight was 63.4 ± 9.1 kg, and their BMI was 23.2 ± 2.4 kg/m2. The mean operative time was 105.5 ± 28.9 min with a mean estimated blood loss of 165.8 ± 75.5 ml. The average follow-up period ranged from 5 to 14 years, and most patients’ follow-ups became irregular after one year. The most common fixed segments were L4/5 (56/66). There was a significant decrease in VAS scores at six months and at the last follow-up, compared to before surgery. In the 6-month follow-up and at the last follow-up, ODI score significantly improved in comparison to preoperatively. Table 2 shows the clinical outcome data of the patients.

**Table 2:** Clinical outcomes

	Scores	P1	P2	P3
**VAS low back pain**		< 0.001	< 0.001	0.13
Pre-surgery	5.94 ± 1.12			
6 months after surgery	1.71 ± 1.05			
Last follow-up	2.17 ± 1.45			
**VAS leg pain**		< 0.001	< 0.001	0.40
Pre-surgery	6.95 ± 1.60			
6 months after surgery	1.58 ± 0.88			
Last follow-up	1.89 ± 1.61			
**ODI**		< 0.001	< 0.001	0.32
Pre-surgery	61.97 ± 8.77			
6 months after surgery	18.41 ± 4.05			
Last follow-up	22.76 ± 13.60			

P1: P value comparing pre-surgery and 6 months after surgery;

P2: P value comparing pre-surgery and final follow-up;

P3: P value comparing 6 months after surgery and final follow-up.

**Fig. 1:**
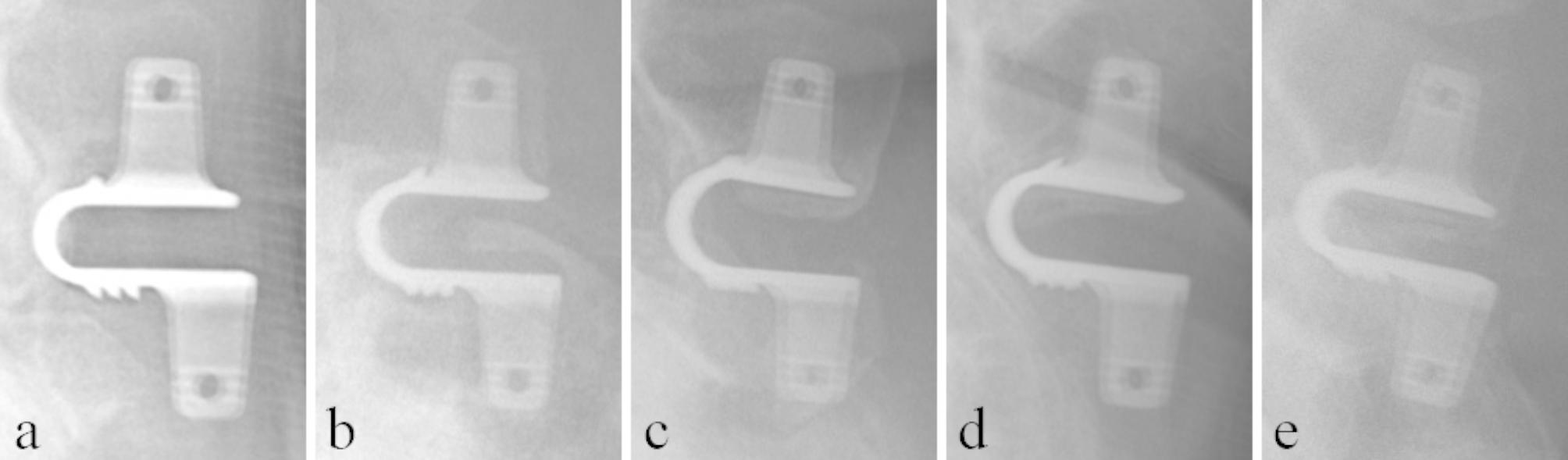
Radiographs of five patients, lateral views. a: No HO. b-e: Various forms of HO in the interspinous space

**Fig. 2:**
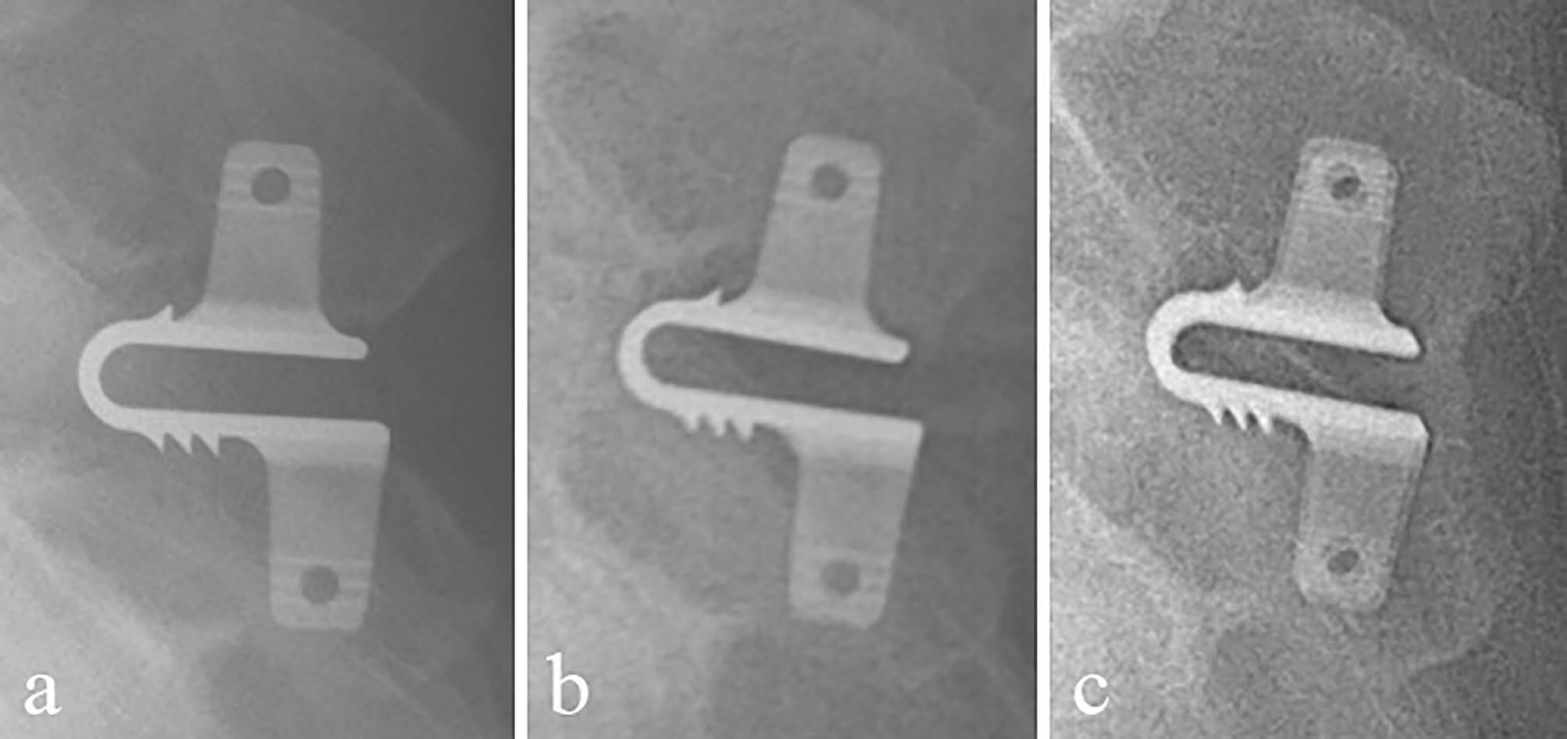
Radiographs of one patient, lateral views. a: No HO seen immediately after surgery. b: Grade 1 HO seen at one year after surgery. c: Interspinous fusion at the final follow-up visit

A total of 59 (89.4%) patients had HO. HO grades were as follows: seven patients with Grade 0 (10.6%), 12 patients with Grade 1 (18.2%), 42 patients with Grade 2 (63.6%), and five patients with Grade 3 (7.6%). ODI score did not differ significantly between high-grade HO (≥ Grade 2) and low-grade HO (< Grade 2) (P = 0.96). Different type of HO and spinous fusion are shown in Figs. 1 and 2, respectively.

**Fig. 3:**
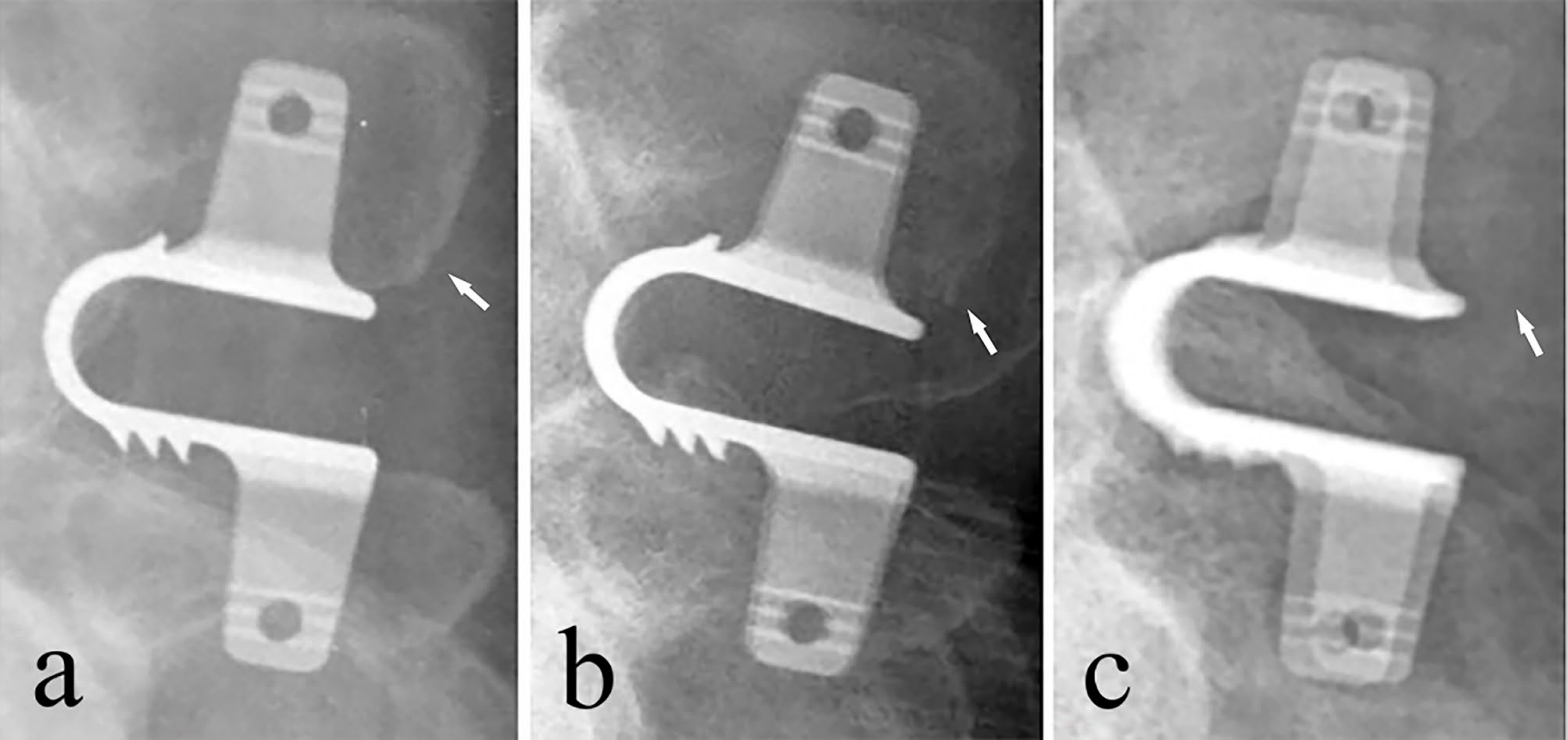
Radiographs of one patient, lateral views. a: Preoperative image. b: Postoperative image at one-year. c: Postoperative image at last follow-up. Arrows indicate osteolysis

Osteolysis occured at the Coflex contact site with the spinous process in 26 (39.4%) patients. ODI score were not significantly different between patients with and without osteolysis (P = 0.68). Figure 3 demonstrates a case of severe osteolysis.

**Fig. 4:**
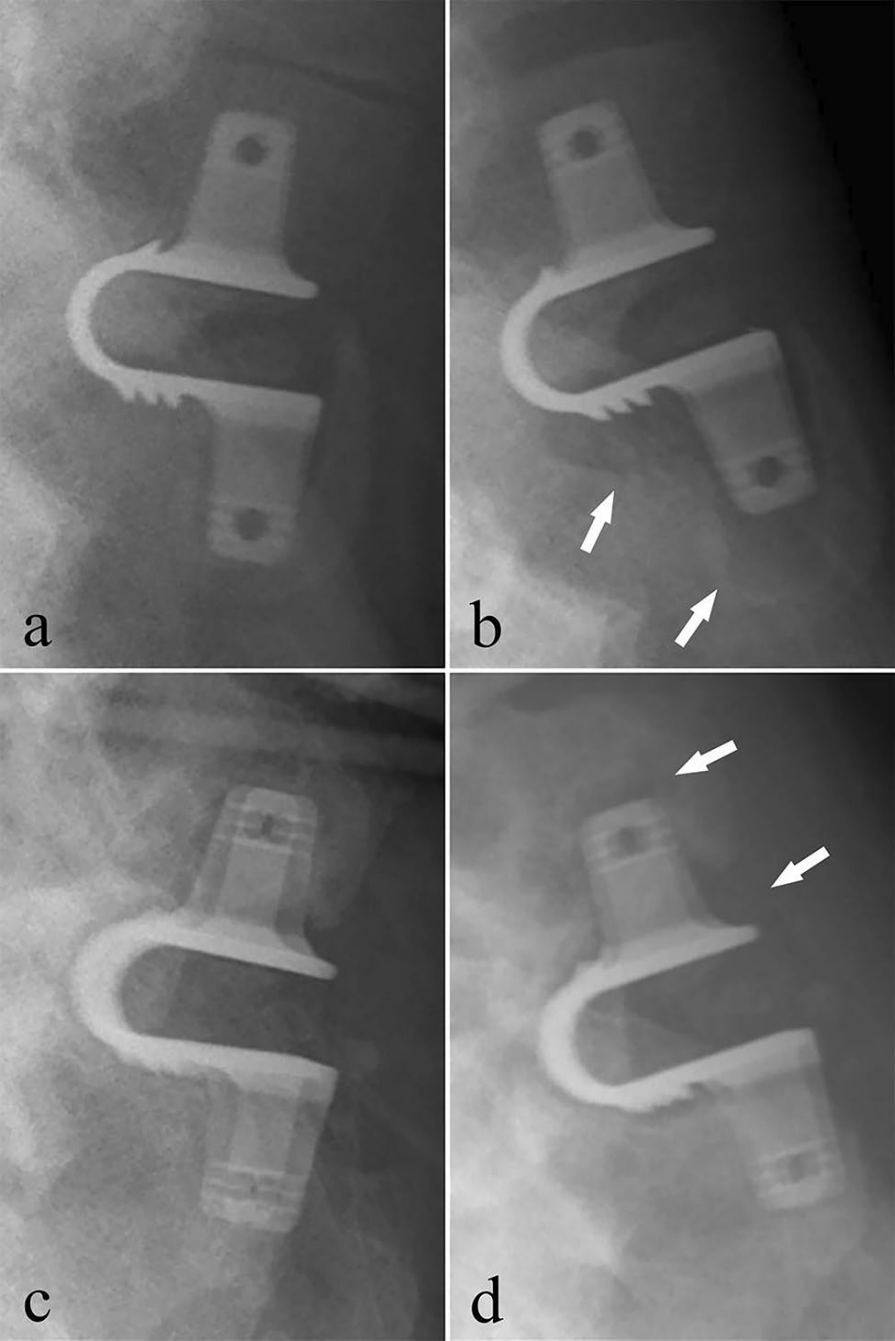
Radiographs of two patients, lateral and over-flexion views. Arrows point out the areas of Coflex loosening in over-flexion position

Coflex loosening was observed in 39 (60%) patients. The following was the specifics: Coflex loosening was found in the upper fixed-wing of eight (20.5%) patients; it was also found in the lower fixed-wing of 12 (30.8%) patients and both upper and lower fixed-wing of 19 (48.7%) patients. There was no significant correlation between the Coflex loosening and the length of follow-up. ODI score did not differ significantly between patients with and without Coflex loosening (P = 0.80). Figure 4 shows various types of Coflex loosening.

**Fig. 5:**
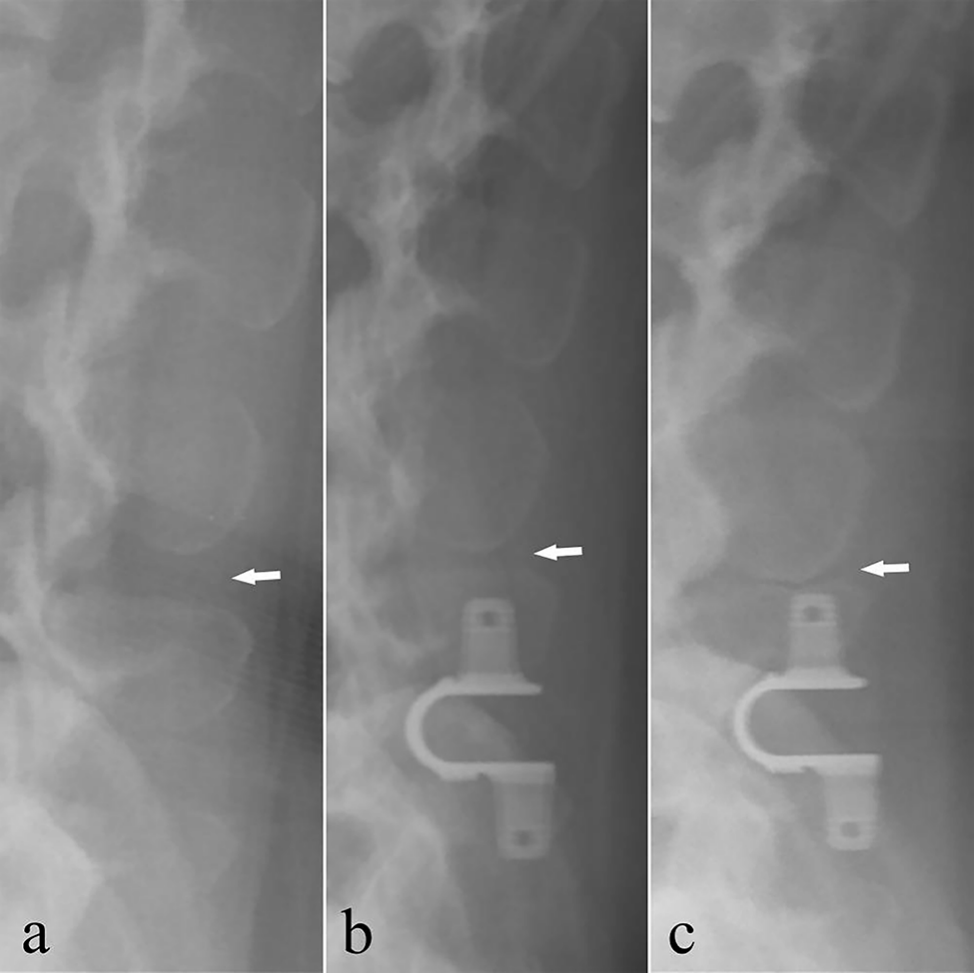
Radiographs of one patient, lateral views. a: Preoperative image. b: Postoperative image at one-year. c: Postoperative image at last follow-up. Arrows point out the gradually approaching adjacent spinous processes

Only two patients had preoperative spinous process anastomosis, which was not relieved or even worsened during follow-up. Spinous process anastomosis was found in 34 (51.5%) patients at different time points during follow-up. Among 34 patients with spinous process anastomosis, 20 (58.8%) had low back pain, while among the 32 patients without spinous process anastomosis, ten (31.2%) had low back pain. There was a statistically significant difference in VAS score for back pain between patients with spinous process anastomosis and patients without spinous process anastomosis (P < 0.05). There was no significant difference in ODI scores between patients with spinous process anastomosis and patients without spinous process anastomosis (P = 0.90). Figure 5 shows the progression of the spinous process anastomosis.

**Fig. 6:**
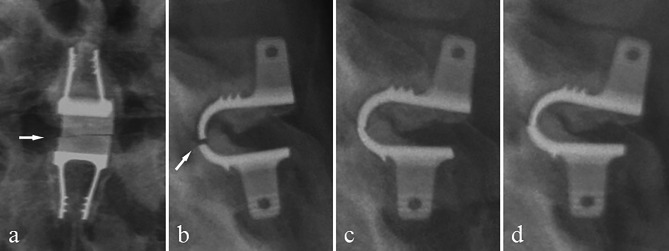
Radiographs of one patient, postoperative image 14 years after surgery. Arrows indicates the fracture

**Fig. 7:**
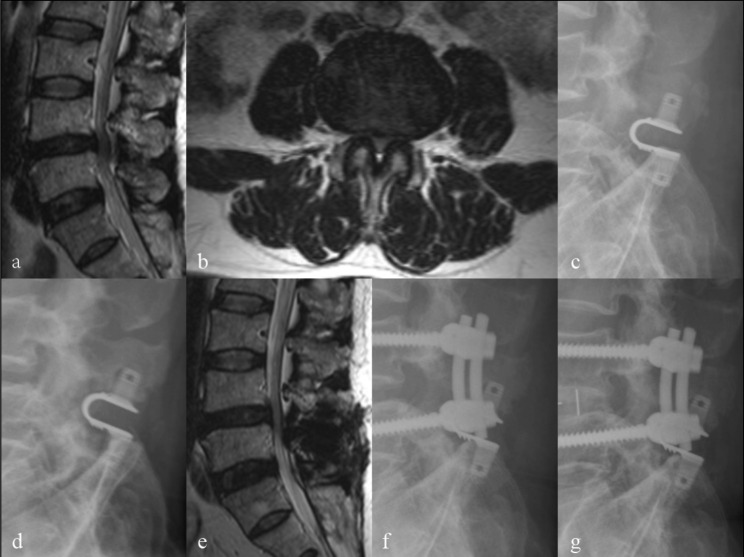
A 52-year-old patient underwent decompression at L4-5 and Coflex fixation at L4-5 for low back pain and lower extremity pain (lumbar spinal stenosis). a-b and c taken preoperative and immediately after surgery respectively. 81 months after the first operation, the patient developed numbness in left hip and lower limb. The X-ray suggested Grade 1 HO and osteolysis at the contact site of the spinous process(d). The MR imaging showed L4-L5 lumbar spinal restenosis(e). He underwent L4-5 TLIF. X ray (f) taken immediately after reoperation. X ray (g) taken 51 months after reoperation

Following a five-year follow-up, one case of device fracture was found (Fig. 6), and nine patients (six males and three females) were identified as having lumbar spinal restenosis based on the criteria. Lower limb numbness in (six patients) was the most common clinical symptom in all patients with restenosis. Total five patients underwent surgical treatment, whereas four patients received conservative treatment. The VAS and ODI scores of all patients who underwent reoperation were significantly improved compared with those before surgery (P < 0.05). Patients who accepted conservative treatments experience symptoms iteratively. Details of all reoperation patients are listed in Table 3. Figure 7 shows a case of reoperation for lumbar spinal restenosis.

**Table 3:** List of patients with Reoperation

Case no.	Age at surgery	Gender	Reoperation interval(months)	Fixation Segment	Initial surgery	Therapeutic strategies	Outcomes
1	41	male	170	L5/S1	L5/S1, D&C	L5/S1, D	Cure
2	52	male	81	L4/5	L4/5, D&C	L4/5, T	Cure
3	54	male	127	L4/5	L4/5, D&C; L5/S1, D	L4/5, D; L5/S1, T	Cure
4	71	female	86	L4/5	L4/5, D&C; L5/S1, D	L3-S1, D; L4/5, T	Cure
5	75	male	79	L4/5	L4/5, D&C	L5/S1, D	Cure

D: decompression; D&C: decompression and coflex fixation; T: transforaminal lumbar interbody fusion (TLIF).

## Discussion

Coflex is a “U”-shaped titanium alloy device, usually placed between adjacent spinous processes following spinal canal decompression. It is designed to maintain the height of the nerve root spinal canal, reduce pressure on the facet joints, and transition from rigid fusion to dynamic fixation while keeping the spine stable [14]. Coflex implantation has definite short and medium-term benefits compared to traditional decompression and fusion surgeries in terms of a short operation time, less intraoperative bleeding, reduced trauma, and faster recovery [15]. However, Coflex also has limitations, such as a higher incidence of adverse events and device failure rates. Furthermore, there is a lack of long-term follow-up and imaging data, and some unique imaging changes and complications have not yet been defined after Coflex implantation.

In this study, HO, a well-known adverse event, was identified as the most frequent imaging change. Tian et al. reported an 81.2% incidence of HO after implanting a Coflex device, which is the first study to determine the probability of HO [13]. Similarly, Haibo Lu et al. reported that the incidence of HO after Coflex device implantation was 42% over five years [16]. However, the incidence of HO after the implantation of a Coflex device was 89.4% in our study, which is higher than the previous reports. This is most likely due to the extended follow-up time. Although, according to ODI score, HO was not found to affect clinical outcomes, HO in the interspinous space can cause rigid fixation of the surgical segment, significantly reducing its range of motion [16]. This study found spinous fusion in five patients, implying that Coflex’s original purpose of long-term dynamic fixation has been lost in these patients. The leading cause of HO after Coflex implantation is unknown; According to the statistical analysis of previous literature, no patient-related or surgical-related factors causing HO after Coflex implantation have been found [16]. We believe that the hollow portion of the U-shaped structure of Coflex combined with the strong osteogenic potential of the spinous process may make it more susceptible to heterotopic bone ingrowth. It could also be related to aseptic inflammation caused by prolonged friction between Coflex and surrounding tissues during daily activities.

Under X-rays, osteolysis at the contact site is visible as an intervening lucent zone between the Coflex and spinous processes. A previous study reported 47% osteolysis between the Coflex and the spinous process at the contact site [17]. In the present study, 39.4% of the cases had osteolysis, consistent with the previous findings. We also observed that all cases of osteolysis resulted in varying degrees of Coflex loosening. Additionally, we found two cases where the spinous processes were almost completely dissolved, leading to complete of Coflex loosening. The majority of osteolysis occurred at the interface between the Coflex and the spinous process, without causing significant damage or progression over time.

It was found that the probability of Coflex loosening is 60%. Previous research has reported Coflex loosening probability of 4.7–20.5% during long-term follow-up [18–21]. Our findings indicated a higher incidence of Coflex loosening and found no effect on clinical outcomes. Most patients with mild loosening have no clinical symptoms, and previous studies have primarily relied on evaluating clinical indicators. Many patients without obvious clinical symptoms are easily overlooked. Notably, that there is no significant correlation between loosening and follow-up time. We suspect that HO is partially ineffective in some cases. Severe HO, particularly around the upper or lower fixed wings, strengthens the connection between Coflex and the spinous process, allowing Coflex to play a fixed role. In addition, the higher incidence of Coflex loosening is of significant importance as it compromises the effectiveness of Coflex in limiting spinal flexion movement and affects its long-term dynamic stabilization function. This provides valuable insights for the improvement of Coflex.

In the previous literature, spinous process anastomosis was frequently described as Baastrup’s disease (BD). It is prevalent among middle-aged and older people with slow progression. It is characterized by hypertrophy of the spinous process as imaging features and often causes low back pain [22–24]. The lateral radiographs of the patients revealed that their spinous processes were consistent with each other and had corresponding bone sclerosis. However, spine hypertrophy was found in only a few patients. Spinous process anastomosis was observed in 34 (51.5%) patients, 16 patients developed within one year of surgery, and the remaining patients developed at various follow-up times until the last follow-up.

We hypothesized that the postoperative spinous process anastomosis results from the Coflex implantation that compresses the adjacent segment’s spinous process, causing them to fit. Furthermore, the collapse of the intervertebral space increased the range of motion of the index level and restoring lumbar spine curvature in the upright position will further fit the adjacent spinous processes, resulting in the emergence of spinous process anastomosis. According to previous research, the incidence of BD in people over 70 years old is increasing. The incidence rate of BD in people over 80 years of age is 81.3% [25]. Age and vertebral degeneration significantly affect the spinal process anastomosis, which is further complicated in the follow-up. The VAS score for back pain differed statistically significantly between patients with and without spinous process anastomosis. Therefore, spinous process anastomosis might have affected the clinical outcome. Furthermore, spinous process anastomosis may exaggerate vertebral degeneration such as spondylolisthesis, facet joint hypertrophy, and cystic lesion on the articulating surface.

The most common long-term complication and the leading cause of Coflex reoperation was lumbar spinal restenosis. We found that the primary causes of restenosis were ligamentum flavum hypertrophy, scar hyperplasia, disc herniation, and articular process cohesion. Maida et al. found patients where large osteophytes intruded into the spinal canal and compressed the dural sac, resulting in recurrent LSS symptoms more than three years after surgery [26]. However, HO was not confirmed as a risk factor for restenosis in our study, and we also did not observe restenosis caused by osteophyte invasion of the spinal canal (RR: 0.94, 95% CI: 0.10–8.88). It could be due to the morphology of HO varies greatly, with only a few specific HO locations causing restenosis.

With a follow-up period of ≥ five years, we found only one case of device fracture and no spinous process fractures or fixed-wing breakage. These complications were associated with surgical technique and bone density, commonly reported immediately after surgery. In the present study, a prosthesis fracture near the center of the Coflex U-shaped structure was observed more than ten years after surgery. We believe that this may be due to device defects and the patient’s long-term lumbar spine movement. However, such case is extremely rare. The overall durability and structural integrity of Coflex have been satisfactory during long-term follow-up.

### Limitations

Our study has several limitations. First, it is a retrospective study with a limited number of participants. Few patients had irregular follow-ups one year after surgery, making it difficult for us to monitor complications and imaging changes. Hence, larger sample sizes studies with regular long-term follow-up should be conducted. Second, the factors influencing these imaging changes have not been thoroughly investigated.

## Conclusion

Our study identified various imaging changes after Coflex implantation, and majority of them did not affect clinical outcomes. The majority of patients had HO, but osteolysis and Coflex loosening were relatively rare. The VAS score for back pain of these patients was higher if they have spinous process anastomosis. After five-year follow-up, we found lumbar spinal restenosis and prosthesis fracture cases.

## Data Availability

The datasets generated and/or analyzed during the current study are not publicly available due data sharing was not included in the informed consent process but are available from the corresponding author on reasonable request.

## References

[1] Lonne G, Fritzell P, Hagg O, Nordvall D, Gerdhem P, Lagerback T, Andersen M, Eiskjaer S, Gehrchen M, Jacobs W, van Hooff ML, Solberg TK (2019). Lumbar spinal stenosis: comparison of surgical practice variation and clinical outcome in three national spine registries. Spine J.

[2] Wang J, Ullah S, Solano MA, Overley SC, Bumpass DB, Mannen EM (2022). Changes in kinematics, kinetics, and muscle activity in patients with lumbar spinal stenosis during gait: systematic review. Spine J.

[3] Lurie J, Tomkins-Lane C (2016). Management of lumbar spinal stenosis. BMJ.

[4] Kurra S, Lavelle WF, Silverstein MP, Savage JW, Orr RD (2018). Long-term outcomes of transforaminal lumbar interbody fusion in patients with spinal stenosis and degenerative scoliosis. Spine J.

[5] Katz JN, Zimmerman ZE, Mass H, Makhni MC (2022). Diagnosis and management of lumbar spinal stenosis: a review. JAMA.

[6] Lu K, Liliang PC, Wang HK, Liang CL, Chen JS, Chen TB, Wang KW, Chen HJ (2015). Reduction in adjacent-segment degeneration after multilevel posterior lumbar interbody fusion with proximal DIAM implantation. J Neurosurg Spine.

[7] Khalaf K, Nikkhoo M (2021). Comparative biomechanical analysis of rigid vs. flexible fixation devices for the lumbar spine: a geometrically patient-specific poroelastic finite element study. Comput Methods Programs Biomed.

[8] Davis RJ, Errico TJ, Bae H, Auerbach JD (2013). Decompression and Coflex interlaminar stabilization compared with decompression and instrumented spinal fusion for spinal stenosis and low-grade degenerative spondylolisthesis: two-year results from the prospective, randomized, multicenter, Food and Drug Administration Investigational device exemption trial. Spine (Phila Pa 1976).

[9] Zhong J, O’Connell B, Balouch E, Stickley C, Leon C, O’Malley N, Protopsaltis TS, Kim YH, Maglaras C, Buckland AJ (2021). Patient outcomes after single-level Coflex Interspinous Implants Versus single-level laminectomy. Spine (Phila Pa 1976).

[10] Zhang Y, Lu D, Ji W, He F, Chen AC, Yang H, Zhu X (2021). Which is the most effective treatment for lumbar spinal stenosis: decompression, fusion, or interspinous process device? A bayesian network meta-analysis. J Orthop Translat.

[11] Xu C, Ni WF, Tian NF, Hu XQ, Li F, Xu HZ (2013). Complications in degenerative lumbar disease treated with a dynamic interspinous spacer (Coflex). Int Orthop.

[12] Kong DS, Kim ES, Eoh W (2007). One-year outcome evaluation after interspinous implantation for degenerative spinal stenosis with segmental instability. J Korean Med Sci.

[13] Tian NF, Wu AM, Wu LJ, Wu XL, Wu YS, Zhang XL, Xu HZ, Chi YL (2013). Incidence of heterotopic ossification after implantation of interspinous process devices. Neurosurg Focus.

[14] Trautwein FT, Lowery GL, Wharton ND, Hipp JA, Chomiak RJ (2010). Determination of the in vivo posterior loading environment of the Coflex interlaminar-interspinous implant. Spine J.

[15] Li AM, Li X, Yang Z (2017). Decompression and coflex interlaminar stabilisation compared with conventional surgical procedures for lumbar spinal stenosis: a systematic review and meta-analysis. Int J Surg.

[16] Y LHH, Y Z, L G, Y L (2020). Heterotopic ossification following decompression and interspinous dynamic stabilization for treating lumbar degenerative disease based on Minimum Follow-Up of five years and X-ray imaging. J Med Imaging Health Inf.

[17] Lee N, Shin DA, Kim KN, Yoon DH, Ha Y, Shin HC, Yi S (2016). Paradoxical radiographic changes of Coflex Interspinous device with Minimum 2-Year Follow-Up in lumbar spinal stenosis. World Neurosurg.

[18] Bae HW, Davis RJ, Lauryssen C, Leary S, Maislin G, Musacchio MJ (2016). Three-year follow-up of the prospective, randomized, controlled trial of Coflex Interlaminar stabilization vs Instrumented Fusion in patients with lumbar stenosis. Neurosurgery.

[19] Zheng X, Chen Z, Yu H, Zhuang J, Yu H, Chang Y (2021). A minimum 8-year follow-up comparative study of decompression and coflex stabilization with decompression and fusion. Exp Ther Med.

[20] Davis R, Auerbach JD, Bae H, Errico TJ (2013). Can low-grade spondylolisthesis be effectively treated by either coflex interlaminar stabilization or laminectomy and posterior spinal fusion? Two-year clinical and radiographic results from the randomized, prospective, multicenter US investigational device exemption trial: clinical article. J Neurosurg Spine.

[21] Aggarwal N, Chow R (2021). Real world adverse events of interspinous spacers using manufacturer and user facility device experience data. Anesth Pain Med (Seoul).

[22] Lin WT, Xie FQ, Lin SH, Yang RB, Shen HW, Cai XF, Chen W, Wang ZY (2021). Full-endoscopic Approach Forchronic Low Back Pain from Baastrup’s Disease: interspinous plasty. Orthop Surg.

[23] Schwartz RH, Urits I, Viswanath O (2019). Extensive degeneration of vertebral body leading to Baastrup’s Disease: a Radiographic Review of an image. Pain Ther.

[24] Filippiadis DK, Mazioti A, Argentos S, Anselmetti G, Papakonstantinou O, Kelekis N, Kelekis A (2015). Baastrup’s disease (kissing spines syndrome): a pictorial review. Insights Imaging.

[25] Kwong Y, Rao N, Latief K (2011). MDCT findings in baastrup disease: disease or normal feature of the aging spine?. AJR Am J Roentgenol.

[26] Maida G, Marcati E, Sarubbo S (2012). Heterotopic ossification in vertebral interlaminar/interspinous instrumentation: report of a case. Case Rep Surg.

